# Pioglitazone counteracts the tumor necrosis factor-α inhibition of follicle-stimulating hormone-induced follicular development and estradiol production in an *in vitro* mouse preantral follicle culture system

**DOI:** 10.1186/1757-2215-6-69

**Published:** 2013-09-30

**Authors:** Shuichiro Hara, Toshifumi Takahashi, Mitsuyoshi Amita, Koki Matsuo, Hideki Igarashi, Hirohisa Kurachi

**Affiliations:** 1 Department of Obstetrics and Gynecology, Yamagata University Faculty of Medicine, Yamagata 990-9585, Japan

**Keywords:** Polycystic ovary syndrome, Pioglitazone, TNF-α, Follicle culture, PPAR-γ

## Abstract

**Background:**

Polycystic ovary syndrome (PCOS) is a common endocrine disorder in women of reproductive age and is characterized by chronic anovulation. Insulin resistance may be a key component of the pathogenesis of this disorder. Pioglitazone is a thiazolidinedione derivative that acts by improving insulin resistance via the peroxisome proliferator-activated receptor-γ (PPAR-γ) pathway. Reportedly, pioglitazone improves the anovulation status in patients with PCOS. In the present study, we examined whether pioglitazone directly affects ovarian follicular development and steroidogenesis using *in vitro* mouse preantral follicle culture system.

**Methods:**

An isolated individual *in vitro* mouse preantral follicle culture was used to test the effects of pioglitazone on the follicle development and steroidogenesis. Tumor necrosis factor-α (TNF-α), which plays a role in insulin resistance, has been reported to inhibit the follicle stimulating hormone (FSH)-induced follicular development and steroidogenesis in an *in vitro* mouse preantral follicle culture system. Therefore, we examined whether pioglitazone counteracts these effects by TNF-α. We assessed the follicle diameter and follicle survival and antral-like cavity formation rates, the 17β-estradiol (E2) levels in the culture medium, and the ovulation rate using the *in vitro* preantral follicle culture.

**Results:**

Pioglitazone treatment counteracted the inhibition of TNF-α in FSH-induced follicle development in a dose-dependent manner. Pioglitazone, at a concentration of 5 μM, which was the minimum effective concentration, significantly counteracted the inhibition of TNF-α in FSH-induced follicle survival (29 versus 56%, *P* < 0.05), antral-like cavity formation (29 versus 48%, *P* < 0.05), E2 concentration in the culture medium (mean ± SEM = 21 ± 1 versus mean ± SEM = 27 ± 1 pg/mL, *P* < 0.05), and human chorionic gonadotropin-induced ovulation rate (9 versus 28%, *P* < 0.05).

**Conclusions:**

Pioglitazone counteracted the inhibition by TNF-α on FSH-induced follicle development and steroidogenesis in the *in vitro* mouse preantral follicle culture. The results suggest that pioglitazone may directly affect the follicular development and steroidogenesis.

## Background

Polycystic ovary syndrome (PCOS) is a common endocrine disorder in women of reproductive age, affecting 5–10% of the population [1], and is characterized by anovulation and infertility. Although the exact pathogenesis of PCOS remains unknown, insulin resistance and compensatory hyperinsulinemia are considered relevant [2]. Hyperinsulinemia contributes to excessive ovarian androgen production and the reduction of the hepatic synthesis of sex hormone-binding globulins, resulting in an elevated level of free androgen [3,4]. Intraovarian hyperandrogenism suppresses araomatase activity and 17β-estradiol (E2) production in granulosa cells [5,6] and proliferation and arresting antral follicle development.

The circulating levels of adipocytokines, such as tumor necrosis factor-α (TNF-α) and interleukin 6, which raise insulin resistance, are higher in obese and nonobese women with PCOS than those in weight matched non-PCOS control women [7,8]. TNF-α is critcically involved in the apoptosis of preantral and antral follicles in mouse and human ovaries [9,10]. Moreover, TNF-α directly affects the ovarian steroidogenesis in granulosa cells in *in vitro* culture [11].

Insulin resistance with compensatory hyperinsulinemia is observed in approximately 70% of patients with PCOS, irrespective of obesity [12,13]. Several studies have suggested that decreases in insulin secretion improve infertility and ovulation [14-16]. Therefore, patients with PCOS are often treated with insulin-sensitizing drugs such as metformin and thiazolidinedione derivatives [17]. Pioglitazone is a thiazolidinedione derivative that has been used for the treatment of type 2 diabetes mellitus. Pioglitazone decreases peripheral insulin resistance via the peroxisome proliferator-activated receptor-γ (PPAR-γ) pathway [18]. Several studies have demonstrated that pioglitazone improves clinical, hormonal, and metabolic status in patients with PCOS [15,19-21]. Moreover, pioglitazone directly inhibits estradiol and testosterone production in human ovarian cells *in vitro*[22]. Pioglitazone-stimulated reduction in peripheral insulin resistance and its direct effect on the ovaries might be effective for inducing ovulation in patients with PCOS. However, the direct effects of pioglitazone on the ovarian follicular development are still unclear.

We previously established the *in vitro* mouse preantral follicle culture system to assess the effects of bezafibrate, a lipid-lowering fibrate, on follicular development [23]. As bezafibrate is a nonselective ligand for PPAR-α, δ, and γ [24,25], we demonstrated that the bezafibrate may exert direct action on the ovarian follicular development and steroidogenesis through the PPAR-γ pathway [23]. Because pioglitazone is a specific PPAR-γ agonist, we hypothesized that pioglitazone may also directly affect the ovarian follicular development and steroidogenesis.

The aim of this study was to examine whether pioglitazone affects the ovarian follicular development and steroidogenesis using an *in vitro* mouse preantral follicle culture system.

## Methods

### Animals

This study was performed with permission from the Committee of Animal Experimentation at the Yamagata University Faculty of Medicine. Mature female ICR mice at 6 weeks or older were used in this study. They were housed in a temperature- and light-controlled room at 23–25°C, on a 12-h light:dark cycle (lights on, 0700–1900 h) and fed pellet food and water ad libitum.

### Reagents

Human pituitary follicle-stimulating hormone (FSH) (catalog no. F4021) and TNF-α (catalog no. T6674), were purchased from Sigma-Aldrich® (St. Louis, MO). Pioglitazone was kindly donated by Takeda Pharmaceutical Company Limited, Osaka, Japan.

### Collection of preantral follicles

Collection of preantral follicles from the mice was reported previously [23]. Briefly, female mice were killed by cervical dislocation, and early preantral follicles were mechanically isolated from the ovaries using a 30-gauge needle under a stereomicroscope. The collected preantral follicles were placed and pooled in *N*-2-hydroxyethylpiperazine-N’-2-ethanesulfonic acid (HEPES)-minimal essential medium (MEM, catalog no. 12360038; Invitrogen Corp, Carlsbad, CA) supplemented with 5% fetal bovine serum (FBS; catalog no. 12603c; JRH bioscience, Lenexa, KS). After washing with HEPES-MEM 2 times, follicles with the following characteristics were selected: (1) diameter, 120–150 μm; (2) immature oocyte centrally located within the follicle; (3) intact basal membrane; and (4) surrounded by theca cells [26]. These procedures were performed at 37°C.

### *In vitro* preantral follicle culture

*In vitro* preantral follicle culture was reported previously [23]. The follicle culture medium consisted of α-MEM GlutaMax™ (catalog no. 32561037; Invitrogen Corp., Carlsbad, CA) supplemented with 5% FBS, 100 units/mL penicillin, 100 μg/mL streptomycin, 5 μg/mL insulin, 5 μg/mL transferrin, and 5 ng/mL selenium (ITS; Invitrogen Corp., Carlsbad, CA). The medium was sterilized with a 20-μm pore filter after the addition of supplements, which resulted in the basal medium. As FSH treatment at 100 mIU/mL was the minimum effective concentration for developing follicles (data not shown), 100 mIU/mL FSH were added to the basal medium in the *in vitro* follicle culture system. Follicles were individually cultured per well in a 24-well plate (Falcon™; Becton Dickinson and Company, Franklin Lakes, NJ) containing 1 mL culture medium at 37°C with 5% CO_2_ in air atmosphere. Follicles were cultured for 13 days. Subsequently, follicular growth was monitored every 2 days (day 2, 4, 6, 8, 10, and 12 of culture) under an inverted microscope at a 200× magnification. Each follicle image was captured by a cooled charge-coupled device camera system (DP-50; Olympus, Tokyo, Japan), and follicle diameter was measured using Image J imaging system software version 1.43 (National Institutes of Health, Bethesda, MD). A total of 500 μL conditioned medium was refreshed with preincubated medium every 2 days (day 2, 4, 6, 8, 10, and 12). The spent media collected on the indicated days were pooled per culture plate and frozen at −80°C for a successive E2 determination. When follicles clearly formed an antral-like cavity on day 12, ovulation and meiotic resumption were induced by refreshing media with culture media supplemented with 5 IU/mL human chorionic gonadotropin (hCG, Mochida Pharmaceutical Co., Tokyo, Japan). On day 13, follicles were checked for ovulation 16 h after hCG administration, and ovulation was considered when the follicle was visually ruptured and the cumulus oocyte complex (COC) extruded from the follicles. The ovulated COCs were evaluated for cumulus expansion and germinal vesicle breakdown (GVBD), indicating meiotic resumption of oocytes, after removing the surrounding cumulus cells by repeated pipetting in the presence of 300 IU/mL hyaluronidase.

### Assessment of follicle survival

Using the method reported by Adriaenssens et al. [27] and Lenie et al. [28], we assessed follicle morphology and classified the follicular development stage as follicular, diffuse, or antral in the *in vitro* preantral follicle culture. Briefly, the follicular stage is characterized by preservation of the follicle basal membrane. Initially, theca cells grow out to the culture plate and granulosa cell proliferation is limited. The follicles remain in this stage until day 4 of culture. The diffuse stage is characterized by marked granulosa cell proliferation and a large preantral follicle. From day 4 to day 6 of culture, granulosa cells overgrow the theca cell monolayer through the basement membrane, and the follicles gradually enlarge. From day 6 or 8 up to day 12 of culture, granulosa cells differentiate into 2 spatially and functionally distinct populations, cumulus cells and mural granulosa cells, and the follicles form an antral-like cavity, which characterizes the antral stage.

In this culture system, surviving follicles were defined as those that could retain their oocyte completely embedded within the granulosa cell mass and that exhibited no signs of degeneration [28,29]. Follicle degeneration was characterized by failure of the granulosa cells to multiply, release oocytes, or by collapse [28].

### 17β-estradiol measurement

Media cultured with individual mouse follicles were replaced every other day, and the collected media were pooled and stored at −80°C until the measurement of E2 levels was performed. E2 levels in the collected culture media were measured using the estradiol enzyme immunoassay kit (Estradiol EIA Kit) (catalogue no. 582251; Cayman Chemical Co., Ann Arbor, MI) according to the manufacturer’s protocol. Culture media from non-surviving follicles were excluded from the experiment.

### Experimental conditions and evaluation parameters

In this experiment, the control group consisted of follicles cultured in media without FSH in the presence of the vehicle. All compounds were added to the culture media during the *in vitro* culture period. TNF-α was dissolved in distilled water at 10 μg/mL and was stored at −80°C until use. Pioglitazone was dissolved in dimethyl sulfoxide (DMSO) at 10 mM and was stocked at −80°C until use. The final concentration of DMSO in the medium never exceeded 0.1%. In the preliminary study, we tested the effects of DMSO as a vehicle on follicle development. There were no significant differences of follicle development between the groups treated with and without DMSO (data not shown).

Effects of various treatments on follicle development and steroidogenesis were evaluated as follows: follicle diameter, follicle survival, antral-like cavity formation, and measurement of E2 concentration in culture media. Moreover, we evaluated the effects of various treatments on ovulation and GVBD, indicating resumption of meiosis.

**Figure 1:**
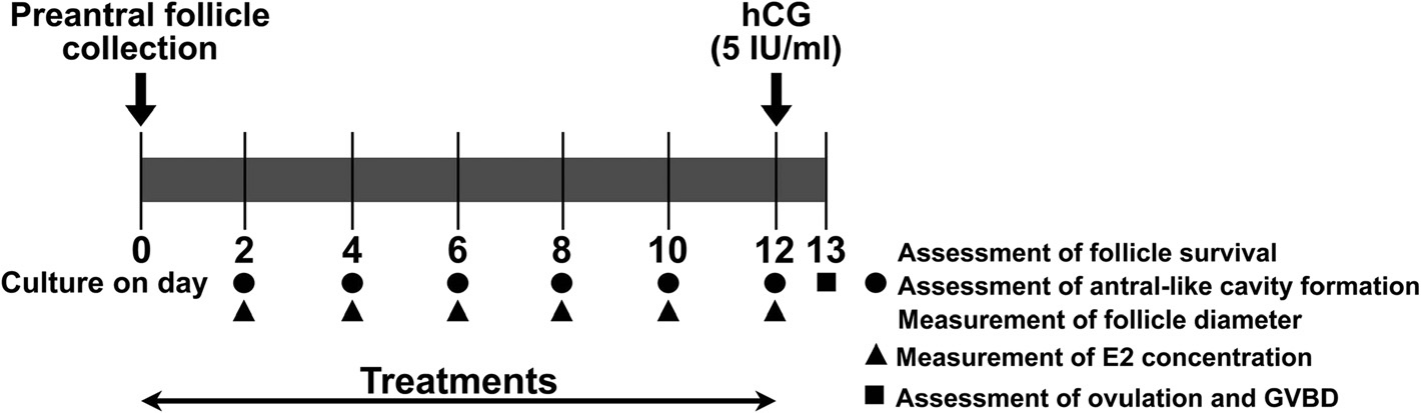
**Study scheme.** Scheme used for *in vitro* culture of mouse follicles for the assessment of follicle diameter, follicle survival, antral-like cavity formation, 17β-estradiol (E2) concentration in the culture media, ovulation, and germinal vesicle breakdown (GVBD) after human chorionic gonadotropin (hCG)treatment is shown. Early preantral follicles were mechanically isolated from mice ovaries as described in Methods. Collected follicles were individually cultured per well in a 24-well plate for 12 days. Follicles were cultured with various treatments. The follicles were inspected for morphology, follicle survival, and antral-like cavity formation every other day as described in Methods. Media were refreshed every other day, and E2 concentrations in the collected media were measured. After 12 days of culture, hCG (5 IU/mL) was added to the culture media to induce ovulation. After 16 h of hCG treatment, ovulation and GVBD were evaluated.

We previously demonstrated that TNF-α significantly inhibited FSH-induced follicular development and steroidogenesis in a dose-dependent manner [23]. In the present study, we examined the effects of pioglitazone on the follicle development and steroidogenesis by using the *in vitro* follicle culture system. Experimental scheme shows in Figure 1.

### Statistical analysis

All experiments consisted of at least 5 independent experimental runs. Differences between means were calculated by one-way ANOVA, followed by a post hoc test, and data expressed as percentages were analyzed by Fisher’s exact probability test using GraphPad Prism version 5.0 for Windows (GraphPad Software Inc., San Diego, CA). Values are given as mean ± SEM. Significant differences are defined as *P* < 0.05.

## Results

**Figure 2:**
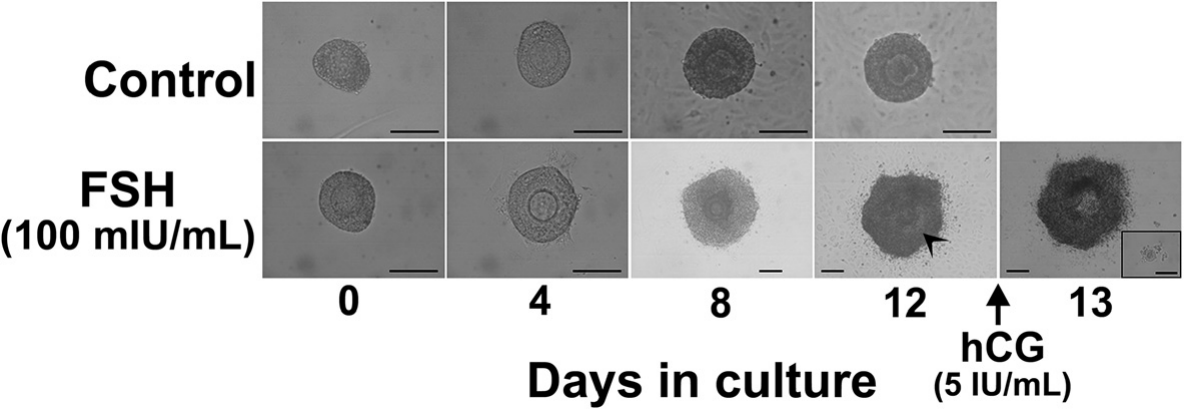
***In vitro *mouse preantral follicle culture system and the effects of FSH on follicular development.** Examples of follicular development treated with or without follicle stimulating hormone (FSH) (100 mIU/mL) are shown. The majority of follicles not treated with FSH (control) grew in a multilayered pattern and did not survive. On the other hand, follicles cultured with FSH (100 mIU/mL) grew larger and formed antral-like cavities at 10 to 12 days of culture. The cumulus oocyte complex was visibly extruded from the antral-like cavity after 16 h of the administration of 5 IU/mL of hCG (inset). Bar = 100 μm.

Our previous data show that 100 mIU/mL of FSH treatment significantly induced the follicle growth and increase in E2 concentration in the culture medium compared to control samples without FSH treatment [23]. While follicles did not grow when cultured without FSH (Figure 2, upper panel), follicles cultured with FSH grew larger and formed an antral-like cavity at 10 to 12 days of culture (Figure 2, lower panel).

TNF-α is a pro-inflammatory cytokine that induces cell death [30]. TNF-α also influences follicle development and steroidogenesis [11]. Further, in patients with PCOS, serum and follicular fluid levels of TNF-α are higher than in patients without PCOS [31,32]. Therefore, we examined the effects of TNF-α on follicular development. In the *in vitro* culture, TNF-α significantly inhibited FSH-induced follicular development and steroidogenesis in a dose-dependent manner with a minimal effective dose of 5 ng/mL. Therefore, we used the *in vitro* preantral follicle culture system with TNF-α as a model to study follicular development of PCOS.

**Figure 3:**
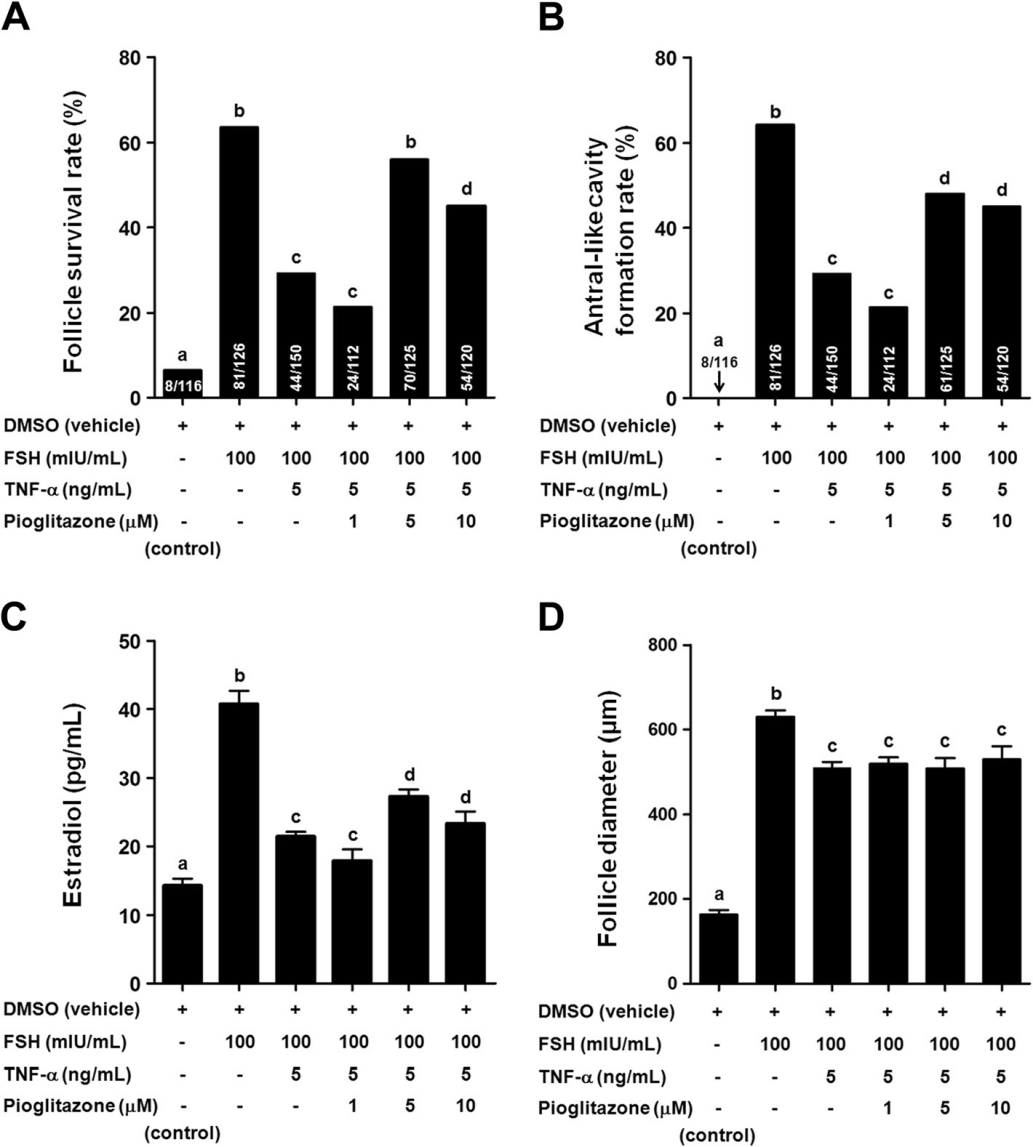
**Pioglitazone counteracts the inhibition of FSH-induced follicular development and steroidogenesis by TNF-α.** Effects of pioglitazone on the inhibition of follicle-stimulating hormone (FSH)-induced follicular development and steroidogenesis by tumor necrosis factor-α (TNF-α). Follicles were cultured with 1, 5, 10 μM of pioglitazone in the presence or absence of 5 ng/mL of TNF-α or 100 mIU/mL of FSH. **A)** Follicle survival rates at 12 days of culture with various treatments are shown. Numbers inside bars indicate the number of surviving follicles/total tested follicles. **B)** Antral-like cavity formation rates at 12 days of culture with various treatments are shown. Numbers inside bars indicate the number of follicles with antral-like cavity/total tested follicles. **C)** 17β-estradiol (E2)concentrations in the culture media at 12 days of culture with various treatments are shown. **D)** Follicle diameters at 12 days of culture with various treatments are shown. Data are shown as the mean ± SEM. The group treated only with dimethyl sulfoxide (DMSO) was used as a control. Bars with different letters represent a significant difference (*P* < 0.05).

We examined whether pioglitazone could counteract the inhibition of FSH-induced follicular development and steroidogenesis by TNF-α. Pioglitazone significantly counteracted the inhibition of FSH-induced follicular development and steroidogenesis by TNF-α in a dose-dependent manner (Figure 3). TNF-α (5 ng/mL) inhibited the FSH-induced follicle survival rate. Although 1 μM of pioglitazone failed to show an effect, both 5 and 10 μM of pioglitazone significantly counteracted the inhibition of FSH-induced follicle survival by TNF-α (Figure 3A). TNF-α inhibited the FSH-induced antral-like cavity formation rate. Although 1 μM of pioglitazone failed to show an effect, both 5 and 10 μM of pioglitazone significantly counteracted the inhibition of FSH-induced antral-like cavity formation rates by TNF-α. TNF-α (5 ng/mL) inhibited the FSH-induced E2 production (Figure 3C). Although 1 μM of pioglitazone failed to show an effect, both 5 and 10 μM of pioglitazone counteracted the inhibition of FSH-induced E2 production by TNF-α (Figure 3C). While TNF-α significantly decreased FSH-induced follicle diameter at 12 days of culture, pioglitazone did not counteract the inhibition by TNF-α in follicle diameter at 12 days of culture (Figure 3D).

We examined the effects of pioglitazone on the hCG-induced ovulation and meiotic resumption of oocytes with or without FSH and TNF-α (Table 1). While TNF-α (5 ng/mL) inhibited the hCG-induced ovulation rate in the FSH-treated group, pioglitazone (5 μM) significantly counteracted the inhibition of the ovulation rates per total tested follicles and follicles with antral-like cavity formation by TNF-α in the FSH-treated groups. All oocytes underwent GVBD after COC ovulation in the FSH-treated groups.

**Table 1:** Effects of pioglitazone on ovulation and meiotic resumption in follicles treated with FSH and TNF-α

**Treatment**	**Number of ovulated follicles/total tested follicles (%)**	**Number of ovulated follicles/number of follicles with antral-like cavity formation (%)**	**Number of GVDB oocytes/number of ovulated COCs (%)**
Control (DMSO)	0/87 (0)^a^	0/0 (0)^a^	0/0 (0)^a^
TNF-α (5 ng/mL)	0/72 (0)^a^	0/0 (0)^a^	0/0 (0)^a^
Pioglitazone (5 μM)	0/80 (0)^a^	0/0 (0)^a^	0/0 (0)^a^
FSH (100 mIU/mL)	60/126 (48)^b^	60/81 (74)^b^	60/60 (100)^b^
FSH + TNF-α (5 ng/mL)	14/150 (99)^c^	14/44 (32)^c^	14/14 (100)^b^
FSH + TNF-α (5 ng/mL) + pioglitazone (5 μM)	35/125 (28)^d^	35/60 (58)^d^	35/35 (100)^b^

FSH, follicle stimulating hormone; TNF-α, tumor necrosis factor-α; DMSO, dimethyl sulfoxide; GVBD, germinal vesicle breakdown; COCs, cumulus oocyte complexes. ^a-d^ Values with different superscript letters within the same column differ significantly. *P* < 0.05.

## Discussion

In the present study, we examined the direct effects of pioglitazone on the follicular development and steroidogenesis by using an *in vitro* mouse preantral follicle culture system. While TNF-α inhibited the FSH-induced follicle development, such as follicle survival, antral-like cavity formation, and E2 production, pioglitazone counteracted these inhibitory effects by TNF-α.

*In vitro* mouse ovarian culture methods provide a useful tool for studying follicle development [23,33-35]. We employed an *in vitro* mouse ovarian follicle culture system for up to 12 days to study the effects of various compounds on FSH-induced follicle development and steroidogenesis. The mechanism of TNF-α inhibition of FSH-induced follicle development and steroidogenesis remains unknown. TNF-α is a well-known cytokine that is capable of inducing apoptosis in cells [30]. TNF-α is also known as adipocytokine that can induce insulin resistance [36]. In the ovary, TNF-α is critically involved in the follicles apoptosis [9,10]. Moreover, TNF-α has direct effects on ovarian steroidogenesis [11,37]. Several studies have reported that TNF-α decreased steroidogenic acute regulatory protein (StAR) mRNA and protein levels [38,39].

In the present study, we showed that pioglitazone counteracted the TNF-α inhibition in FSH-induced follicle development and steroidogenesis in an *in vitro* culture system. The precise mechanisms of restoration of TNF-α inhibition of FSH-induced follicle development and steroidogenesis by pioglitazone were unknown. Recently, we examined whether bezafibrate, a ligand for PPARs (α, δ, γ) rescued the TNF-α inhibition of follicular development and steroidogenesis [23]. Our previous data showed that PPAR-γ was expressed in mouse preantral follicles and that GW1929, a selective PPAR-γ agonist, counteracted the inhibition of FSH-induced follicular development by TNF-α. Furthermore, GW9662, a selective PPAR-γ antagonist, canceled the restorative effects of bezafibrate on the inhibition of FSH-induced follicular development and steroidogenesis by TNF-α. Therefore, we suggested that, among the three PPARs, PPAR-γ is important for follicular development. In fact, it is reported that deletion of PPAR-γ results in impaired fertility in genetically targeted mice [40].

It is well known that thiazolidinediones, such as pioglitazone and rosiglitazone, specifically bind to PPAR-γ, which is located on macrophages, adipose tissue or human granulosa lutein cells [18,41]. Pioglitazone is a thiazolidinedione derivative that acts by improving insulin resistance via the PPAR-γ pathway. Pioglitazone improves peripheral insulin resistance, ovulation, and hyperandrogenism in women with PCOS [42-44]. Seto-Young et al. reported that pioglitazone has a direct action on the human ovary [22]. Kim et al. showed that pioglitazone decreased the follicular fluid levels of TNF-α in patients with PCOS [45]. Moreover, several reports have demonstrated that PPAR-γ activation reduced the expression of TNF-α and other inflammatory cytokines *in vivo*[46,47]. Pioglitazone may act on PPAR-γ and reduce TNF-α. However, the detailed pathway through which pioglitazone suppresses TNF-α is unclear. Further examinations are needed to determine the detailed mechanism by which pioglitazone affects follicular development.

## Conclusions

Pioglitazone counteracted the inhibition of FSH-induced follicular development and steroidogenesis by TNF-α. These results suggest that pioglitazone may directly act on ovarian functions through PPAR-γ activation in patients with PCOS.

## Abbreviations

PCOS: Polycystic ovary syndrome; PPAR-γ: Peroxisome proliferator-activated receptor-γ; TNF-α: Tumor necrosis factor-α; FSH: Follicle stimulating hormone; E2: 17β-estradiol; hCG: Human chorionic gonadotropin; HEPES: *N*-2-hydroxyethylpiperazine-N’-2-ethanesulfonic acid; MEM: Minimal essential medium; COC: Cumulus oocyte complex; GVBD: Germinal vesicle breakdown; DMSO: Dimethyl sulfoxide.

## Competing interest

The authors declare that they have no competing interests.

## Authors’ contributions

SH carried out the experiment of follicle culture and drafted the manuscript. TT participated in the design of the study and drafted the manuscript. MA carried out the measurement of E2. HI performed the statistical analysis. HK participated in its design and coordination and helped to draft the manuscript. All authors read and approved the final manuscript.
